# Rapid axial roll test outperforms alternative positional tests in identifying the affected ear in HSC-BPPV: an observational cohort study

**DOI:** 10.3389/fneur.2024.1432608

**Published:** 2024-06-19

**Authors:** Juanli Xing, Yanning Yun, Shu Zhang, Pan Yang, Xiongxiong Bai, Leyi Zhang, Ming Zhang

**Affiliations:** ^1^Department of Otorhinolaryngology Head and Neck Surgery, The First Affiliated Hospital of Xi'an Jiaotong University, Xi’an, China; ^2^Department of Otorhinolaryngology Head and Neck Surgery, The Affiliated Hospital of Inner Mongolia Medical University, Hohhot, Inner Mongolia, China; ^3^Department of Neurology, Jingyang County Hospital, Xianyang, China; ^4^Department of Thoracic Surgery, The First Affiliated Hospital of Xi’an Medical University, Xi’an, China; ^5^Department of Humanity, University of Amsterdam, Amsterdam, North Holland, Netherlands; ^6^Department of Medical Imaging, The First Affiliated Hospital of Xi'an Jiaotong University, Xi’an, China

**Keywords:** HSC-BPPV, rapid axial roll test, alternative positional tests, supine roll test, affected semicircular canal

## Abstract

**Purpose:**

To evaluate the utility of supine roll test (SRT) and alternative positional tests, such as head-shaking test (HST), seated supine positioning test (SSPT), bow and lean test (BLT), and rapid axial roll test (RART) in determining the affected semicircular canal of horizontal semicircular canal benign paroxysmal positional vertigo (HSC-BPPV).

**Methods:**

In an observational cohort study, 553 patients diagnosed with HSC-BPPV were divided into five groups in terms of different positional tests received: SRT group (*n* = 110), HST+ SRT (*n* = 112), BLT + SRT (*n* = 114), SSPT+SRT (*n* = 108) and RART+SRT (*n* = 109). The same method was used for the last four groups: The patients were first subjected to different alternative positional tests and then to SRT, and the nystagmus was observed separately to determine the affected side. The primary outcomes compared included the accuracy and sensitivity of these tests in the determination of the affected semicircular canal in HSC-BPPV.

**Results:**

Patients with nystagmus elicited by positional tests accounted for 84.99% (470/553). The elicitation rate of nystagmus of SRT was lowest, being 77.27% (85/110). The elicitation rate of nystagmus were higher in the test groups than in the control group, and RART+SRT group yielded the highest elicitation rate of nystagmus (95.41%, 104/109). Among the alternative positional tests, RART attained the highest elicitation rate of nystagmus (101/109, 92.66%). Comparison between alternative positional tests and SRT, RART and SRT showed obviously better agreement in determining the affected semicircular canal (85.45%, 96/109) and eliciting nystagmus (95.41%, Kappa = 0.642), but no difference was found in curative effect when the affected side was accurately determined (χ^2^ = 1.618, *p* = 0.655).

**Conclusion:**

All alternative positional tests are helpful for eliciting nystagmus in patients with HSC-BPPV, and the significant advantages of RART include high-sensitivity in eliciting nystagmus and high accuracy in determining the affected semicircular canal, which provided objective support for the correct diagnosis of HSC-BPPV and the successful reduction of otolith.

## 1 Introduction

Benign paroxysmal positional vertigo (BPPV), represents the most prevalent peripheral vestibular vertigo disorder (1). Mechanistically, BPPV results when loose otoliths detached from the utricle and enter the semicircular canal. The condition tends to present as transient vertigo concomitant with the change of head position along the gravitational force. Posterior semicircular canal BPPV (PSC-BPPV) was most common, with an incidence of approximately 60–90% (2). Next comes horizontal semicircular canal BPPV (HSC-BPPV), its incidence standing somewhere between 16 and 31% (3). Canalith repositioning procedures (CRPs) have long been a successful and most important treatment for BPPV and are seen as the gold standard for the management of BPPV. The success rate of CRPs in treating PSC-BPPV is over 90%, against the rate of HSC-BPPV at 60 to 90%.

SRT was first proposed by Pagninil in 1989 (4). Since then, the clinical diagnosis of HSC-BPPV has been essentially based on SRT. With BPPV, determination of the affected semicircular canal is of paramount importance for a successful repositioning maneuver. Until now, the SRT remains the most accurate test method for HSC-BPPV, but its accuracy may change with different clinical settings. Nonetheless, SRT is still subject to multiple limitations. First, the maximal range of neck movement is no more than 75°, and the angle and speed of the movement have to be within certain range when the head turns from side to side. Secondly, due to the “velocity storage” mechanism, patients with HSC-BPPV may suffer from nausea and vomiting during the test, resulting in most patients refusing to cooperate, leading to test failure. Moreover, neck rotation not only increases the burden of the cervical spine, but also tends to cause iatrogenic injury. Therefore, because of the complexity and directional variability of nystagmus in HSC-BPPV, and many limitations of SRT, the elicitation rate of nystagmus is low. Failure to determine the affected ear may well render the treatment of HSC-BPPV difficult.

To address the limitations of SRT, attempts have been made to use alternative positional tests, including pseudo-spontaneous nystagmus (PSN) (5), head-shaking test (HST) (6), seated-supine positioning test (SSPT), or “lying-down positioning test (7), bow and lean test (BLT) (8), among others. Multiple studies demonstrated that these positional tests seemed unable to accurately predict the affected semicircular canal in HSC-BPPV patients (8–10), but capable of helping clinicians to determine the affected semicircular canal. In fact, elicitation rate of nystagmus of the aforementioned positional tests was low. Additionally, these procedures were complicated, time-consuming, and labor-intensive, and frequent alternative positional tests might increase the suffering of the patients (11). Therefore, it is urgent to work out a new method for the identification of affected semicircular canal in HSC-BPPV patients.

RART is performed by turning the body of a patient 90° to either side while the patient maintains a straight line from head to toes in a supine position with a 30° head elevation. The advantage of RART lies in its ability to quickly turn over in an axial direction at a rate of 90°/s, thereby achieving the maximal acceleration, so that the otolith moves a greater distance within the long arm of the horizontal semicircular canal with endolymph fluid flowing toward or away from the ampulla, minimizes the burden of cervical vertebra, and avoids iatrogenic injury. RART is especially appropriate for those who are obese, short-necked and have limited mobility. Therefore, the rapid axial roll test (RART) (12) proposed by the author’s team in the early stage can fully address the limitations of SRT in determining the affected semicircular canal.

Therefore, in the present study, we compared the elicitation rate of nystagmus among HST, BLT, SSPT, RART and SRT, and examined the sensitivity and accuracy of different positional tests in identifying HSC-BPPV, and the agreement in determining the affected ear and eliciting nystagmus between SRT and other tests.

## 2 Materials and methods

### 2.1 Study design and participants

The project has been approved by the Ethics Committee of the First Affiliated Hospital of Xi’an Jiaotong University (reference NO. 2021-1560), and all participants were informed in detail about the purpose, process and method of the study, and signed informed consents before enrollment.

In a prospective cohort study within the positional test, 553 patients who had been diagnosed with HSC-BPPV at the Vertigo Disease Center of the First Affiliated Hospital of Xi’an Jiaoting University from September 1st, 2020 and September 30th, 2022, were included.

The inclusion criteria, which were based on standardized protocols, were as follows: (1) Patients suffering from transient vertigo induced by turning over (the BPPV diagnostic criteria of the Bárány Society in 2015) (13); (2) Patients presenting horizontal geotropic or apogeotropic nystagmus lasting less than 1 min; (3) Unilateral onset with no contraindications for CRP; and (4) Patients who could receive treatment and cooperate during follow-ups. Patients who had one of the following conditions were excluded from the study: (1) Central vertigo; (2) Psychogenic vertigo; (3) Cervical vertigo; (4) Coexisting inner and middle ear diseases; (5) vestibular migraine, Meniere’s disease, etc.; (6) Severe cardiovascular and cerebrovascular diseases; and (7) Poor compliance.

### 2.2 Study protocol

All patients who were enrolled into this study underwent a detailed history interrogation and physical examination, to rule out middle-ear, inner-ear, and other vestibular disorders or central nervous system pathologies. The following tests or examinations were performed, including physical examinations, otoscopy, pure-tone audiometry, pure-tone audiometry and tympanometry, video nystagmography (VNG), video head impulse test (vHIT), vestibular-evoked myogenic potentials (VEMP), and imaging studies like CT or MRI.

553 patients diagnosed as HSC-BPPV were divided into five groups in terms of positional tests received: SRT (*n* = 110), HST + SRT (*n* = 112), BLT + SRT (*n* = 114), SSPT+SRT (*n* = 108) and RART+SRT (*n* = 109) group. The researchers utilized the identical operative technique in last four groups: The patients were first subjected to other positional tests and then to SRT, the nystagmus was separately observed. In order to ensure accurate diagnosis and the homogeneity of all participants who took part in this study, experienced attending doctors who received the same training conducted HST, BLT, SSPT, RART, and SRT on all patients on an infrared video electronystagmograph (ZT-VNG-II by Zhiting Medical Instruments, Shanghai. Co., Ltd.), or video goggles. The nystagmus characteristics were also recorded in detail.

**Figure 1:**
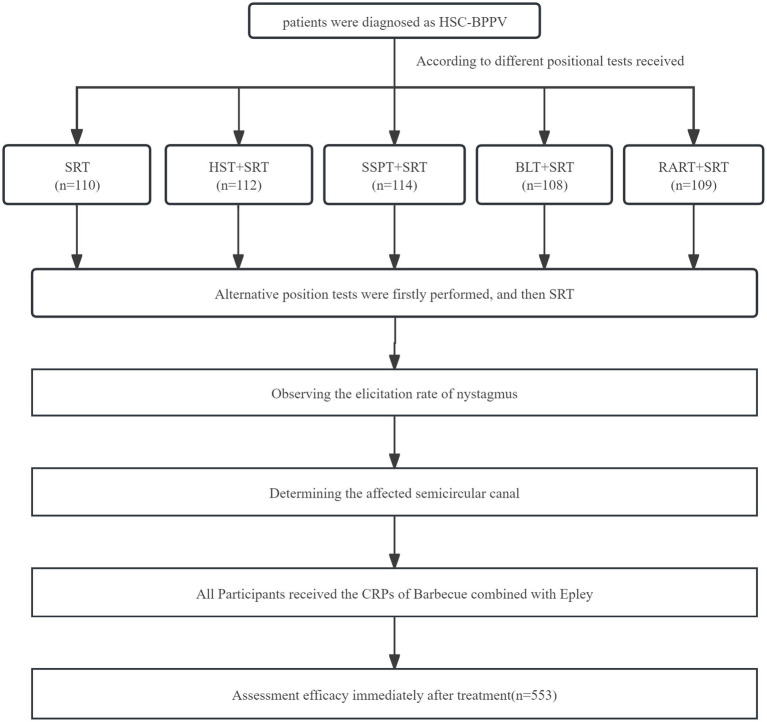
Flow diagram.

Patients received the CRPs (Barbecue and Epley therapy) performed by a skilled physiotherapist (Xing JL). Immediately after CRPs, the physiotherapist also conducted the Dix-Hallpike, RART and an anterior canal provoking test to observe positional nystagmus, with the final results analyzed. The flow chart is shown in Figure 1.

### 2.3 Nystagmus recording

By using the video goggles, parameters of nystagmus were comprehensively and meticulously recorded, including direction (geotropic and apogeotropic horizontal nystagmus), latency (time from head movement initiation to nystagmus onset), duration (time from onset to cessation of nystagmus), and nystagmus intensity (peak value of slow phase angular velocity, measured in °/s).

### 2.4 Alternative positional tests

#### 2.4.1 Head-shaking test

In a seated position with a 30° head tilt forward, the head of subjects was passively shaken horizontally no more than 30°, at a frequency of 2 Hz. Nystagmus after rapid head-shaking, or “head-shaking nystagmus (HSN),” was observed via video goggles, with relevant parameters recorded, including nystagmus direction, slow phase angular velocity, and duration. An intensity exceeding 2°/s with a minimum of 5 successive nystagmus beats indicated a positive result (14).

#### 2.4.2 Bow and lean test

Subjects were required to tilt head down 90 degrees swiftly, and then up 45 degrees to 60 degrees, and the nystagmus features were recorded, including bowing nystagmus (BN) and leaning nystagmus (LN) (15). Distinct categories of HSC-BPPV were identified on the basis of the relationship between nystagmus and head movement. The affected semicircular canal was on the same side of BN, opposite to the LN in the case of cupulolithiasis of the horizontal canal. On the contrary, the affected semicircular canal was on the same side of LN, opposite to the BN in the case of canalolithiasis of the horizontal canal.

#### 2.4.3 Seated supine positioning test

With SSPN, also known as lying-down nystagmus (LDN), participants switched from sitting position to supine position with a 30° head elevation, and this positional change induced SSPN (16).

When the patient shifted from a seated to a supine position, according to Ewald’s second law, the otoliths were in the postbrachium of the semicircular canal, and it were away from the ampulla, evoking inhibitory excitation. However, if the otoliths were near the single cruz or forearm, otoliths moved toward the ampulla, eliciting excitatory nystagmus.

#### 2.4.4 Rapid axial roll test

The subject, wearing video goggles, laid supine on a 30° inclined pillow. A examiner fixed the subject’s forehead with one hand and the shoulder with the other, and helped the patient to execute a rapid horizontal axis flip at 90°/s^2^, in the following order: supine → left-ear-down → supine → right-ear-down → supine position.

#### 2.4.5 Supine roll test

Subjects equipped with video goggles laid supine on a 30-degree inclined pillow and was asked to quickly turn head to right or left at 90 degrees, to identify the affected semicircular canal based on the parameters of nystagmus.

hc-BPPV-ca had the following characteristics: There was geotropic nystagmus when the ear of patient was downward; the direction of nystagmus in supine position pointed to the weak side and duration of nystagmus was less than 1 min. The intensity of nystagmus was usually stronger with the head turned to the affected ear in the SRT or RART.

hc-BPPV-cu was characterized by the following features: There was apogeotropic nystagmus when the ear of patient was downward; the direction of nystagmus in supine position pointed to the weak side and duration of nystagmus lasted more than 1 min. The intensity of nystagmus was usually weaker with the head turning to the affected ear in the SRT or RART.

### 2.5 Treatment methods

A uniform treatment approach was employed, involving two cycles of Barbecue and one cycle of Epley on an SRM-IV vertigo diagnostic system. The cure rate of patients after CRPs was analyzed.

#### 2.5.1 Barbecue

Each patient was placed on and fixed to an SRM-IV automatic swivel chair, and performed a sequential series of positions, including sitting, supine, affected-ear-down, supine, unaffected-ear-down, and prone, affected-side-down position, ending up with a return to the sitting position. Each position lasted for 30 s or until the disappearance of nystagmus or vertigo, to ensure a comprehensive and effective CRP.

#### 2.5.2 Epley

With Epley, the patient was placed on and fixed to the/an SRM-IV automatic swivel chair, turned right/left 45°, then quickly leaned back at 120° (rotated 120° clockwise/counterclockwise), then turned right/left two times at 90° each. Finally, the automatic swivel chair was slowly returned to the starting position. The parameters of nystagmus in various positions were observed and recorded through the wireless video goggles, the patient remained at each position for 120 s, or until nystagmus and dizziness disappeared for 30–60 s before proceeding to the next step.

For patients who did not respond favorably to the initial CRP, multiple RARTs were administered to reassess the affected semicircular canal. Subsequently, they were subjected to CRP, involving two cycles of the Barbecue and one cycle of the Epley. Patients who were persistently unsuccessful CRP would be given personalized interventions.

### 2.6 Statistical methods

Statistical analysis was conducted by using SPSS 25.0 software package. A *p* < 0.05 was considered to indicate statistically significant difference. Categorical variables were presented by frequency and percentage. Comparison of count data between different groups employed the Chi-square test. When multiple groups were compared, the level of significance was adjusted. Kappa statistics were used to evaluate the agreement between the alternative positional tests and SRT in eliciting positional nystagmus. Kappa value greater than 0 indicated more agreement than expected, and Kappa value less than 0 was indicative of less agreement than expected by chance (17). We rated the agreement level in terms of Kappa value: 1 = perfect agreement; −1 = total disagreement; 0 = agreement fully by chance; 0–0.2 = slight agreement; 0.2–0.4 = fair agreement; 0.4–0.6 = moderate agreement; 0.6–0.8 = substantial agreement; and 0.8–1 = almost perfect agreement (18).

## 3 Results

A total of 553 patients diagnosed as HSC-BPPV were considered for inclusion into the study. No subjects withdrew, and all were included in the final evaluation. We compared basic characteristics of patients in five groups, including sex, age, body mass index (BMI), duration of dizziness and the affected ear and type of BPPV. The clinical data are listed in Table 1. Figure 1 is the Consort flow diagram.

**Table 1:** Baseline characteristics of HSC-BPPV patients.

Characteristic	All participants *n* = 553	SRT *n* = 110	HSN + SRT *n* = 112	BLT + SRT *n* = 108	SSPT+SRT *n* = 114	RART+SRT *n* = 109	X^2^	*p*-value
Gender (*n*, %)							6.983	0.137
Male	175 (31.65)	38 (34.55)	32 (28.57)	32 (29.63)	29 (25.44)	44 (40.37)		
Female	378 (68.35)	72 (65.45)	80 (71.43)	76 (70.37)	85 (74.56)	65 (59.63)		
Age (*n*, %)							1.279	0.865
<40y	83 (15.01)	20 (18.18)	16 (14.29)	14 (12.96)	17 (14.91)	16 (14.68)		
> = 40y	470 (84.99)	90 (81,82)	96 (85.71)	94 (87.04)	97 (85.09)	93 (85.32)		
BMI (*n*, %)							4.129	0.389
<24 kg/m^2^	219 (39.60)	44 (40)	49 (43.75)	48 (44.44)	38 (33.33)	40 (36.70)		
≥24 kg/m^2^	334 (60.40)	66 (60)	63 (56.25)	60 (55.56)	76 (66.67)	69 (63.30)		
Duration of dizziness (*n*, %)							9.947	0.269
<7 days	171 (30.92)	31 (28.18)	23 (20.54)	41 (37.96)	39 (34.21)	37 (33.94)		
7–30 days	252 (45.57)	52 (47.27)	57 (50.89)	45 (41.67)	51 (44.74)	47 (43.12)		
>30 days	130 (23.51)	27 (24.55)	32 (28.57)	22 (20.37)	24 (21.05)	25 (22.94)		
The affected semicircular canal and type of BPPV (*n*, %)							20.187	0.064
R-HSC-Can	282 (50.99)	51 (46.36)	57 (50.89)	48 (44.44)	63 (55.26)	63 (57.80)		
L-HSC-Can	156 (28.21)	37 (33.64)	30 (26.79)	35 (32.41)	32 (28.07)	22 (20.18)		
R-HSC-Cup	61 (11.03)	16 (14.55)	17 (15.18)	10 (9.26)	10 (8.77)	8 (7.34)		
L-HSC-Cup	54 (9.76)	6 (5.45)	8 (7.14)	15 (13.89)	9 (7.90)	16 (14.68)		

HST + SRT, head-shaking test + supine roll test; BLT + SRT, bow and lean test + supine roll test.

SSPT + SRT, seated supine positioning test + supine roll test; RART + SRT, rapid axial roll test + supine roll test.

### 3.1 Sociodemographic/clinical characteristics

Table 1 shows the baseline demographics and clinical characteristics of the participants (*n* = 553). The mean age of all the participants was 55.97 (SD = 13.40) years (range, 18–86). Most of them were females (*n* = 378, 68.35%). The mean BMI of all the participants was 24.45 (SD = 1.83) kg/m^2^. Most of HSC-BPPV were right canalolithiasis (*n* = 282, 50.99%). No significant differences were observed in the sociodemographic/clinical features among the five groups, indicating a balanced and comparable baseline characteristics (relative homogeneity) among the five patient groups.

### 3.2 The elicitation rate of nystagmus in different positional tests

Table 2 exhibits the elicitation rate of nystagmus in five patient groups: SRT group (85/110, 77.27%), HST + SRT group (91/112, 81.25%), BLT + SRT group (93/114, 81.58%), SSPT+SRT (90/108, 83.33%) and RART+SRT (104/109, 95.41%). The elicitation rate of nystagmus was significantly higher in RART+SRT group than in other groups. Among the five groups, there were statistically significant differences in the elicitation rate of nystagmus (χ^2^ = 12.090, *p* = 0.017). Pairwise comparison between groups revealed a statistically significant difference between RART+SRT and the other groups.

**Table 2:** The elicitation rate of nystagmus in different positional tests.

Groups	Cases (*n*)	Cases with elicited nystagmus (*n*)	Elicitation rate of nystagmus (%)	χ2	*p*-value
SRT	110	85	77.27^a^	15.189	*p* = 0.004
HST + SRT	112	91	81.25^a^		
BLT + SRT	114	93	81.58^a^		
SSPT+SRT	108	90	83.33^a^		
RART+SRT	109	104	95.41^b^		
Total	553	470	84.99		

HST + SRT, head-shaking test + supine roll test; BLT + SRT, bow and lean test + supine roll test.

SSPT + SRT, seated supine positioning test + supine roll test; RART + SRT, rapid axial roll test + supine roll test.

a\b: The same letters meaned there was no statistically significant difference between the groups. Different letters meaned there was a statistical significance between the groups. Pairwise comparison between groups and adjust the inspection level. a = 0.05, ****p ≤ 0.005. RART + SRT vs. SRT: χ2 = 15.239, *p* < 0.001; RART + SRT vs. HST + SRT: χ2 = 10.674, *p* = 0.001; RART + SRT vs. BLT + SRT: χ2 = 10.353, *p* = 0.001; and RART + SRT vs. SSPT + SRT: χ2 = 8.354, *p* = 0.004.

### 3.3 The elicitation rate of nystagmus with alternative positional tests and SRT and the cure rate of HSC-BPPV

The patients were first subjected to various alternative positional tests and then SRT. The elicitation rate of nystagmus in each of alternative positional tests and SRT was separately compared (Table 3). RART+SRT yielded the highest elicitation rate of nystagmus, and the difference was statistically significant (alternative positional tests: χ^2^ = 85.558, *p* < 0.001; SRT: χ^2^ = 26.935, *p* < 0.001), and pairwise comparison was used to compare the elicitation rates of nystagmus in alternative positional tests, and the result revealed statistically significant differences between RART and the other three tests, and between BLT and SSPT (χ^2^ = 17.264, *p* < 0.001). Pairwise comparison was utilized to compare the elicitation rates of nystagmus in the test groups where SRT was performed, and statistically significant differences were found between the groups. In terms of the sensitivity and accuracy in identifying the affected side of HSC-BPPV, RART+SRT group had better agreement in determining affected semicircular canal (85.45%), and the differences were statistically significant among test groups (χ^2^ = 103.034, *p* < 0.001). However, there were no significant differences in cure rates of HSC-BPPV between the test groups.

**Table 3:** The elicitation rates of nystagmus for alternative positional tests and SRT and the cure rates of HSC-BPPV.

Groups	Elicitation rates of nystagmus in alternative positional tests (*n*, %)	Elicitation rates of nystagmus in SRT (*n*, %)	Agreement in determining affected semicircular canal (*n*, %)	Cure rate (*n*, %)
HST + SRT	44 (36.61)^a^	89 (79.46)^a^	36 (32.14)	35 (97.22)
BLT + SRT	45 (39.47)^a^	79 (69.30)^b^	30 (26.32)	28 (93.33)
SSPT+SRT	55 (50.93)^a^	96 (88.89)^c^	50 (46.30)	48 (96.00)
RART+SRT	101 (92.66)^b^	102 (93.58)^c^	96 (85.45)	94 (97.92)
χ^2^	85.558	26.935	103.034	1.618
*p*	<0.001	<0.001	<0.001	0.655

HST + SRT, head-shaking test + supine roll test; BLT + SRT, bow and lean test + supine roll test.

SSPT + SRT, seated supine positioning test + supine roll test; RART + SRT, rapid axial roll test + supine roll test.

a\b\c: The same letters meaned there was no statistically significant difference between the groups. Different letters meaned there was a statistical significance between the groups. Pairwise comparison between groups and adjust the inspection level. a = 0.05, ****p ≤ 0.0083.

Elicitation rates of nystagmus in alternative positional tests: RART vs. HST: χ^2^ = 69.747, *p* < 0.001; RART vs. BLT: χ^2^ = 69.727, *p* < 0.001; RART vs. SSPT: χ^2^ = 46.757, *p* < 0.001.

Elicitation rates of nystagmus in SRT: HST + SRT vs. BLT + SRT: χ^2^ = 3.061, *p* = 0.008; BLT + SRT vs. SSPT + SRT: χ^2^ = 12.754, *p* < 0.001; HST + SRT vs. RART + SRT: χ^2^ = 9.379, *p* = 0.002; BLT + SRT vs. RART + SRT: χ^2^ = 21.488, *p* < 0.001.

### 3.4 The agreement between SRT and alternative positional tests

Kappa value was used to assess the agreement between alternative positional tests and SRT in the induction of nystagmus (Table 4). Results showed that there existed a substantial agreement between SRT and RART in terms of the elicitation of nystagmus (95.41% agreement, Kappa =0.642, *p* < 0.001).

**Table 4:** The agreement between SRT and alternative positional tests.

Method		SRT (*n*, %)		Agreement (*n*, %)	Kappa value	*p*-values
		(+)	(−)			
HST (*n*, %)	(+)	39 (34.82)	5 (4.47)	57 (50.89)	0.128	0.053
	(−)	50 (44.64)	18 (16.07)			
BLT (*n*, %)	(+)	34 (31.48)	11 (10.19)	52 (48.14)	0.037	0.633
	(−)	45 (41.67)	18 (16.67)			
SSPT (*n*, %)	(+)	52 (45.62)	3 (2.63)	73 (64.04)	0.295	0.071
	(−)	38 (33.33)	21 (18.42)			
RART (*n*, %)	(+)	99 (90.83)	2 (1.83)	104 (95.41)	0.642	<0.001
	(−)	3 (2.75)	5 (4.59)			

SRT, supine roll test; HST, head-shaking test; BLT, bow and lean test; SSPT, seated supine positioning test; RART, rapid axial roll test.

## 4 Discussion

In this single-center, an observational cohort study, 553 patients diagnosed with HSC-BPPV were enrolled and analyzed. The subjects were from one of the largest general tertiary hospital in Western China. In this study, we found that RART attained the highest elicitation rate of nystagmus (92.66%), and the highest accuracy in determining the affected side (85.45%). Moreover, RART and SRT had substantial agreement in the elicitation of nystagmus (95.41%), and all alternative positional tests were conducive to SRT in eliciting positional nystagmus.

In fact, the intensity of nystagmus triggered by positional tests depends on the number, size, density of the otoliths, the angle between the semicircular canal plane and the direction of gravity, the moving distance of otolithic debris, the angular acceleration and the amplitude of head motion during the positional tests (19, 20). Some scholars reported that they employed SRT alone to identify the affected side of HSC-BPPV, and its accuracy rate was 62% ~ 75% (21). In this study, the elicitation rate of nystagmus with SRT was 77.27% (Table 2), which was a little higher than previously reported findings. Based on our data (Table 2), the elicitation rate of nystagmus was higher in the test groups than in the control group, and RART+SRT group had a higher elicitation rate of nystagmus.

Our comparison showed that RART was more effective than other alternative positional tests in eliciting nystagmus, and the elicitation rate of nystagmus with SRT was increased after different alternative positional tests (Table 3), indicating that the alternative positional tests can help SRT elicit nystagmus. Comparison within the alternative positional test groups exhibited that RART had high-sensitivity in eliciting nystagmus (92.66%), while in ensuing SRT, the elicitation rate of nystagmus was highest (93.58%). RART+SRT group had better agreement in determining affected semicircular canal. The main reason for higher sensitivity in eliciting nystagmus and high accuracy in determining the affected semicircular canal was that the head and neck spine were in a straight line and the positional change along the body axis was very rapid (at an acceleration of 90°/s^2^) during the RART, the maximum effective pulse stimulation was achieved, thereby extending the distance of the otolith movement. This could move those otoliths that could not completely move due to cantonation, adhesion, stenosis of the semicircular canal, or without sufficient driving force. The endolymph fluid in the semicircular canal moves toward or away from the ampulla, causing the cupula to deflect, thus producing the characteristic nystagmus, which helps clinicians to quickly and accurately identify the affected semicircular canal. Table 4 showed there existed a substantial agreement between SRT and RART in terms of the elicitation of nystagmus (95.41% agreement, Kappa =0.642, *p* < 0.001). For obese, short-necked or elderly patients, RART can effectively avoid iatrogenic injury, improve elicitation rate of nystagmus, and increase curative effect. Therefore, we advocate that HSC-BPPV was diagnosed by RART instead of SRT.

Kamei et al. proposed the concept of HST in 1964, and the positive rate of HSN was as high as 86%. HSN in BPPV might partially result from the otolith movements related with endolymph dynamics. Lee et al. observed that half of patients (83/173, 48%) with HC-BPPV and approximately 32% of patients with PC-BPPV had HSN (6, 22). In this study, the elicitation rate of HSN was 36.61%, which was consistent with that reported in a previous study (Table 3). A retrospective study on HSN in the HSC-BPPV patients indicated that sensitivity of HSN was only 30.0% (27/90), and otolithic debris in the semicircular canal usually did not exert a significant impact on the fluid dynamics of the endolymph during head-shaking, which may explain the low sensitivity of HSN in our research (9). In this study, HST and SRT had a low agreement in determination of the affected semicircular canal and only 32.14% (Table 3) and 50.89% had a marginal agreement in terms of the elicitation of nystagmus (Kappa = 0.128) (Table 4). The finding indicated that the sensitivity of HST was low and HST should not be used for the identification of the affected semicircular canals in BPPV.

BLT was first put forward by Choung in 2006. Since then, the effectiveness of the BLT has been discussed in many studies (15), but its sensitivity seems to be controversial. Some researchers believe that BLT is not a perfect test for identifying the affected ear of HSC-BPPV. Choung et al. reported that bowing and leaning nystagmus could be observed in 88.56% (23 of 26) patients, and suggested BLT was of clinical significance as a diagnostic tool for HSC-BPPV (15). Lee et al. reported that the positive rate of BLT was 62.9% (56 of 89) in hc-BPPV and 41.3% (31 of 75) in hc-BPPV-cu, and BLT may become a method of choice if the remission rate of HSC-BPPV could be further elevated (23). Kim et al. reported that only in 55% of hc-BPPV patients was bowing and leaning nystagmus detected, and BLT might not be a very useful method in lateralizing the hc-BPPV (10). In this study, approximately 39.47% (45/114) of patients showed BN or/and LN, which was lower than previously reported. Meanwhile Table 3 shows that BLT and SRT had the lowest agreement (merely 26.32%) in the determination of affected semicircular canal, and Table 4 exhibits that BLT and SRT had a slight agreement in the elicitation of nystagmus (52/114, 45.61%, Kappa = 0.037), suggesting this method might often fail in identifying the affected side. One possible reason was the incongruence between the anatomical structure of HSC and the head pitching movement (24). All in all, we believe BLT is not a very helpful test in determining the affected side of the semicircular canal.

SSPT was first brought up by Nuti in 1996, and its positive rate stood somewhere between 38.2 and 75.9% (16). In general, SSPN points to the healthy side in hc-BPPV-ca and to the affected side in hc-BPPV-cu. However, widely varying findings were reported regarding the elicitation rate and nystagmus characteristics of SSPN in recent years. In 2006, Han et al. reported that only 58 (38.2%) of 152 cases with SHC-BPPV were found to have SSPN (25). Califano et al. systematically examined the significance and utility of “secondary localization signs” in the diagnosis of BPPV in 2010, and concluded that SSPN induced by SSPT had the highest elicitation rate (26). In 2014, Oh et al. reported that SSPN was induced in only 34 (68.0%) of 50 cases of HSC-BPPV, and only in 61.3% did SSPN point to the healthy side and in 78.9%, it pointed to the affected side, so they questioned the value of SSPN in the diagnosis/identification of the affected canal in HSC-BPPV patients (27). In 2020, Martellucci et al. found that 81.34% of patients had SSPN, and they believed that SSPT was of limited value in the laterality identification of HSC-BPPV (8). In this study, the elicitation rate of nystagmus by SSPN was 55 (50.93%), which was only higher than 38.2% reported by Han and lower than the rates of other reports. Table 3 shows that SSPT and SRT had an agreement of 46.30% in determining the affected semicircular canal, and Table 4 shows that SSPT and SRT had a fair consistency in the elicitation of nystagmus in 64.04% (Kappa = 0.295). The main reason was that the pitch motion plane of SSPN was inconsistent with the anatomic plane of the horizontal semicircular canal.

Asprella introduced a concept of “strategy of the minimum stimulus,” and clinically, the treatment option of “minimum stimulation” has a guiding significance, and “high efficiency” and “low incidence of adverse reactions” are a trend of BPPV treatment in the future (28). In this study, RART turned out to be the best test method, with high acceleration, good safety and sensitivity. Moreover, and its elicitation rate of nystagmus, and the accuracy in determining the affected side were higher, and also lent support of the idea of “minimum stimulation.”

## 5 Limitations

Although a large sample size and a wide range of practical application value were an advantage of this paper, this study had some weaknesses. First, this study had a large sample size and many groups, and multiple comparisons may lead to false positive results. Second, the duration of dizziness of HSC-BPPV is longer in this study, which might result in a selection bias toward new patients. Third, this paper is a single-center study. Future multi-center, prospective cohort studies can further verify the findings and come to a conclusion.

## 6 Conclusion

In summary, RART is the most accurate tool for identifying the affected side of HSC-BPPV. RART out-performs SRT in that RART is simple, convenient and safe. These advantages can help avoid iatrogenic injury, effectively induce the characteristic nystagmus, accurately identify the affected semicircular canal, and improve the cure rate of HSC-BPPV. HST, BLT, and SSPT cannot be used alone in the diagnosis of HSC-BPPV. Especially SRT or RART should be used in combination when nystagmus is not clear. Our results provided a more objective reference for clinicians in the diagnosis of with HSC-BPPV.

## Data availability statement

The original contributions presented in the study are included in the article/supplementary material, further inquiries can be directed to the corresponding author.

## Ethics statement

The studies involving humans were approved by the Xi’an Jiaotong University Ethics Committee. The studies were conducted in accordance with the local legislation and institutional requirements. The participants provided their written informed consent to participate in this study.

## Author contributions

JX: Funding acquisition, Methodology, Writing – review & editing. YY: Methodology, Writing – original draft. SZ: Data curation, Writing – review & editing. PY: Data curation, Writing – review & editing. XB: Formal analysis, Writing – review & editing. LZ: Writing – original draft. MZ: Methodology, Writing – review & editing.
